# Simultaneous Larva Migrans and Larva Currens Caused by *Strongyloides stercoralis*: A Case Report

**DOI:** 10.1155/2013/381583

**Published:** 2013-02-17

**Authors:** Liliam Dalla Corte, Mariana Vale Scribel da Silva, Paulo Ricardo Martins Souza

**Affiliations:** Santa Casa de Misericórdia de Porto Alegre, Porto Alegre, RS, Brazil

## Abstract

Strongyloidiasis is an infectious disease caused by the *Strongyloides stercoralis* larvae, which penetrate the skin, go through the lymphatic circulation, and migrate to the lungs before reaching the intestines. They mature and may cause cutaneous strongyloidiasis, known as larva currens because of the quick migratory rate of the larva. The authors describe a case in which the larvae did not follow their natural lymph route, and after penetrating into the intertriginous area, they migrated to the dermis, developing larva migrans in the early phase, and later associated with the typical lesions of larva currens. The diagnosis was confirmed by the presence of larva in the skin biopsy.

## 1. Introduction

*Strongyloides stercoralis* is especially endemic in many tropical countries, where it has become a matter of concern in the public health area [1–7]. The special characteristic features of its life cycle are chronicity, autoinfection, and easy dissemination. It is a parasitic disease difficult to diagnose as it requires the direct visualization of the larvae [1].

The infection is asymptomatic in most cases; however, the pathognomonic skin lesion of chronic strongyloidiasis is the larva currens [2, 3]. Its diagnosis may be difficult to obtain due to atypical manifestations [3].

In this study, we described a rarely observed case of simultaneous larva migrans and larva currens caused by *Strongyloides stercoralis*.

## 2. Case Report

**Figure 1:**
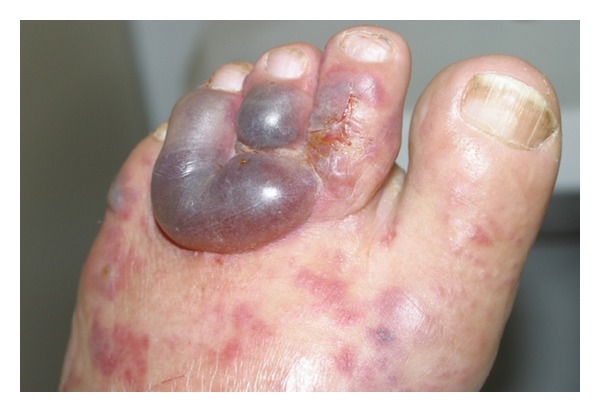
Area larvae penetration. Hemorrhagic blisters in the area: the larvae penetrated the skin and the purpuric serpiginous lesions in the back of the left foot.

**Figure 2:**
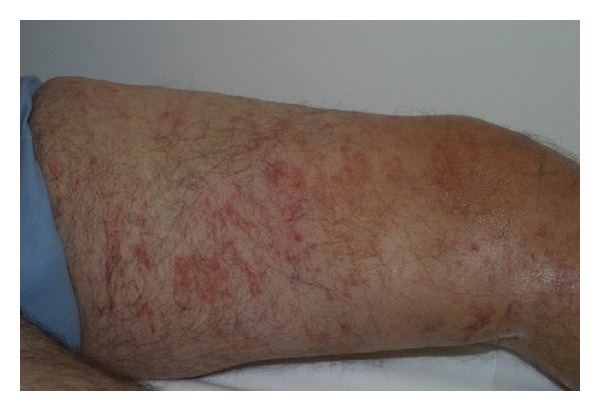
Larva migrans. Diffuse purpuric lesions showing the various routes the larvae took in the left thigh.

**Figure 3:**
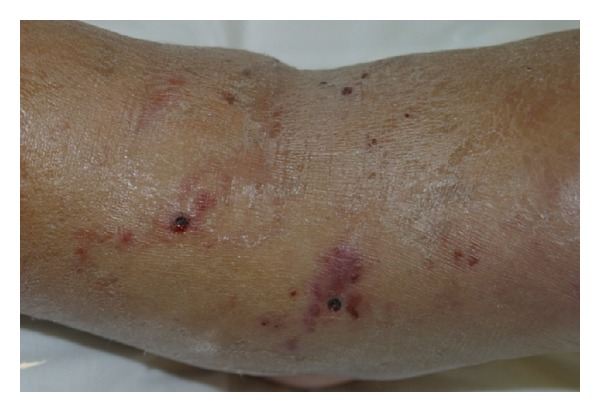
Biopsy site in the popliteal region.

A healthy 36-year-old white man from the city of Porto Alegre, RS, Brazil, was fishing barefoot near a pond and after a period of 24 hours developed some cutaneous hemorrhagic blisters on his left foot (Figure 1). In 3 days, such condition evolved into pruritic, linear to serpiginous, erythematous urticarial lesions in the left lower limb and reached his left thigh (Figures 2 and 3). Progressively, some new urticarial, linear lesions appeared in his trunk. He denied using any medications and did not report any signs or symptoms.

**Figure 4:**
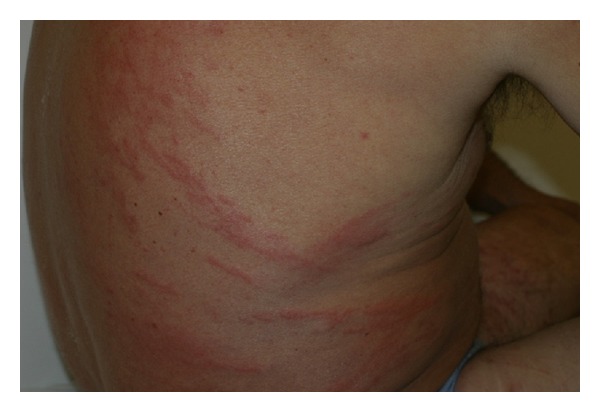
Larva currens. Erythematous, edematous urticarial lesions in the back and the abdomen.

**Figure 5:**
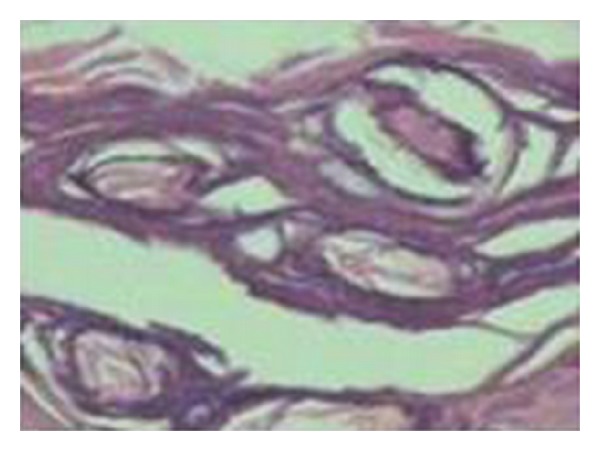
Pathology. PAS 10x: presence of larva PAS positive in stratum corneum.

**Figure 6:**
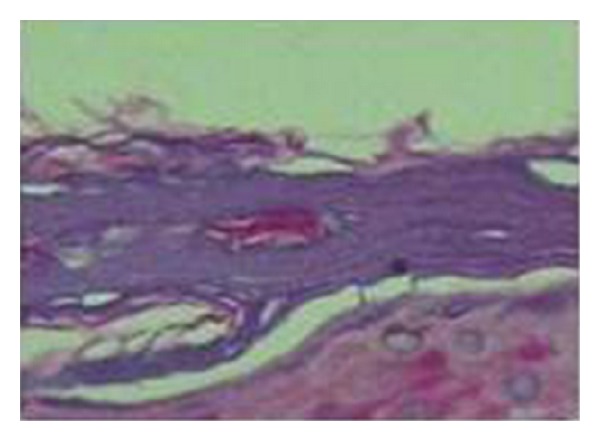
*Strongyloides stercoralis*. Presence of larva in the corneal layer.

There were no changes in the blood, stool, and partial parasitological urine tests, except for the eosinophilia in the blood test. The initial diagnosis was vasculitis due to the purpuric lesions; however, with the emergence of some lesions in the trunk, there was the chance that it could be larva currens. A systemic, empirical treatment was carried out with ivermectin (200 mcg/kg/day) for 3 consecutive days. The skin lesions and the eosinophilia regressed. Later, the pathology showed the larvae in the epidermis (Figures 4, 5, and 6).

## 3. Discussion

This is a patient with a rare and an atypical cutaneous strongyloidiasis, which was confirmed by the presence of larvae in the skin biopsy. The most common form of transmission of *Strongyloides stercoralis* is through the skin, primarily through the feet of people who do not wear any type of shoes [1–3]. The main skin changes occurred due to mechanical, traumatic, toxic, irritant, and antigenic actions from females, larvae, and eggs [3, 4].

After penetrating the dermis, the larvae reach the blood circulation. These larvae may not find their natural route and remain in the integument, thus exhibiting a rarely observed condition of linear dermatitis of larva migrans caused by *Strongyloides stercoralis* [5, 6].

There is some light skin reaction in the site where the penetration of the infective larvae occurred. On reinjection, there is a hypersensitivity reaction that causes edema, erythema, itching, and hemorrhagic and urticarial papules [3, 4, 6]. Sometimes, a single or multiple migration of *Filaroides* larvae is observed in the subcutaneous tissue, resulting in a pruritic, linear to serpiginous, erythematous urticarial lesion characterized as larva currens, more commonly found in the trunk, buttocks, perineum, groin, and thighs, moving at a rate of 5–15 cm/h [5].

The clinical diagnosis is difficult, considering that approximately 50% of cases present no symptoms. The presence of diarrhea, abdominal pain, and urticaria is suggestive. Eosinophilia and serological and radiological findings can be helpful [5].

The parasitological methods or direct examination is based on the combination of the *Strongyloides stercoralis* evolutionary forms [1, 2, 5, 6].

Treatment of strongyloidiasis caused by nematode larvae can be very difficult. The drugs of choice are thiabendazole 50 mg/kg twice daily for 2 to 3 days, albendazole 400 mg once daily for 3 days, and ivermectin 200 mcg/kg single oral dose [7].

This is a case of simultaneous larva migrans in the lower limbs and larva currens in the abdomen and the back, evidencing the change of direction taken by the larva, which was proven by the occurrence of *Strongyloides stercoralis* in the skin biopsy. The early diagnosis allowed an immediate and appropriate therapeutic treatment to avoid greater morbidity.
